# Prospective Risk and Protective Factors for Suicide Attempts Among Black Adolescents Seeking Emergency Department Services

**DOI:** 10.1016/j.jaacop.2025.11.002

**Published:** 2025-11-24

**Authors:** Nadia Al-Dajani, Amanda Jiang, David A. Brent, Jacqueline Grupp-Phelan, T. Charles Casper, Polly Y. Gipson Allen, Cheryl A. King, Pediatric Emergency Care Applied Research Network (PECARN)

**Affiliations:** a University of Louisville, Louisville, Kentucky; b University of Michigan Medical School, Ann Arbor, Michigan; c University of Pittsburgh, Pittsburgh, Pennsylvania; d University of California San Francisco, San Francisco, California; e University of Utah School of Medicine, Salt Lake City, Utah

**Keywords:** adolescence, Black youth, risk assessment, suicide

## Abstract

**Objective:**

Suicide rates among Black youth have been rising at an alarming rate, with a 54% increase between 2018 and 2022. This study investigated sociodemographic, suicide-related, clinical, and interpersonal risk and protective factors of lifetime (cross-sectional) and 6-month (prospective) suicide attempt in a large sample of Black youth who visited the emergency department.

**Method:**

Data from 1,719 Black youths (girls = 1,046 [61%], mean [SD] age =14.98 [1.66] years) were obtained from the Emergency Department Screening for Teens at Risk for Suicide (ED-STARS) Study 1 cohort recruited from 13 nation-wide pediatric emergency departments (Pediatric Emergency Care Applied Research Network [PECARN]). Among 784 youths selected for follow-up interviews based on level of suicide risk, 616 (78.6%) participated in 3- and/or 6-month follow-up interviews. Univariable logistic regression models examined cross-sectional and longitudinal relations between sociodemographic, suicide-related, and other clinical and interpersonal risk and protective factors and suicide attempt. A multivariable logistic regression model predicting follow-up suicide attempt was examined.

**Results:**

Cross-sectional and follow-up univariable models found that gender identity, sexual minority status, suicidal ideation/behavior, nonsuicidal self-injury, hopelessness, depression, agitation, anxiety, sexual abuse history, level of impairment, connectedness (family, peer, school), and impulsivity predicted suicide attempt. The final multivariable model included lifetime suicidal behavior, baseline suicidal ideation in the past week, impulsivity, and all forms of connectedness (area under the curve = 0.91, 95% CI 0.87-0.95) as predictors of suicide attempt, although impulsivity and social/school connectedness were nonsignificant predictors that were retained for improved fit indices.

**Conclusion:**

Our findings highlight the importance of suicide-related factors, hopelessness, functional impairment, sexual and gender minorities, and connectedness at baseline in predicting future suicide attempts in Black youth. Future work should incorporate race-related risk and protective factors.

## Plain language summary

Suicide rates among Black youth are climbing at an alarming rate. This study examined which array of factors predicted suicide attempts 3-6 months later among 784 Black youth who visited the emergency department through follow-up interviews. Results identified several factors that predicted suicide attempts, including sexual and gender minority identity, prior suicidal experiences, and feeling hopeless, amongst others. Feeling connected to family, peers, and school reduced the likelihood of attempting suicide.

The suicide rate among Black youth in the United States increased 54% between 2018 and 2022, with the rate among Black youth surpassing that of White youth for the first time in 2022.^1^ Moreover, high school–age Black youth were the only adolescent racial/ethnic group with documented increases in the rates of both suicide attempts and injury related to suicide attempts (a prominent risk factor for suicide deaths) between 1999 and 2017.^2^ Recent national data from the Youth Risk Behavior Survey indicate that, among Black high school students, 21.6% have seriously considered suicide, and 14.5% have attempted suicide within a 12-month period.^3^

Given these upward trending rates in suicide attempts and suicides, there is an urgent need to address the paucity of research and resultant knowledge gap regarding suicide risk among Black youth. A report released by the Congressional Black Caucus of the US House of Representatives, “Ring the Alarm: The Crisis of Black Youth Suicide in America,”^4^ summarized key epidemiological and clinical findings regarding suicide among Black youth and called for greater prioritization of research to investigate this increase. This prioritization was recently emphasized by an issue brief published by the Substance Abuse and Mental Health Services Administration.^5^

Despite the limited number of studies of suicide risk among Black adolescents, the extant research literature provides some important information.^6^, ^7^, ^8^, ^9^ Social support has been associated with lower levels of depression and suicide risk among Black adolescents in the United States, serving as a protective factor.^2^ Family relationship problems,^6^ recency and frequency of alcohol use,^9^ history of trauma or physical maltreatment, depression, delinquent behavior, and access to lethal means^4^ have all been identified or put forth as risk factors. In a broad sample of racial and ethnic minority youth in the United States, perceived racial discrimination, self-identification as a sexual minority, being a victim of cyberbullying, sadness or hopelessness, and poor mental health (during the COVID-19 pandemic) have also been associated with suicidal behavior.^10^

The extant research literature on youth suicide risk reflects a significant disparity and gap in our knowledge, particularly concerning short-term risk for suicide attempts in Black adolescents. Most studies of youth suicide risk have sampled predominantly European American youth or a broad population of youth characterized by several races and ethnicities. Of studies with mixed race samples, most have not conducted analyses by race. This is a critical gap as the constellation of risk factors or risk phenotype may be different and unique for Black adolescents as well as for the diversity of demographic subgroups within this population. In addition to the possibility of unique cultural, historical, and structural risk factors for Black adolescents, the psychiatric indicators of suicide risk may differ, as almost half of Black adolescents who attempted suicide in 1 investigation had never met criteria for a psychiatric diagnosis before their suicide attempt.^11^ Further, Black adolescents were significantly less likely than White youth to have received a diagnosis of a mental health disorder before death by suicide.^12^^,^^13^ In another investigation, compared with European American girls, Black adolescent girls were found to engage in suicidal behavior with lower levels of depression severity.^14^ Finally, in a large study of 8- to 12-year-old patients who visited a pediatric emergency department (ED), Black preadolescents were less likely to report suicidal thoughts, although lifetime suicide attempt history reported by these preadolescents was similar to other patients.^15^

Given the wide array of contributors to suicide risk, it is imperative that an examination of prospective suicide risk consider factors that span multiple arenas. Although not specific to Black youth, prior work has shown the importance of sociodemographic factors, such as sexual and gender minority identity,^16^ parental education,^17^ and poverty^18^; suicide-related factors, such as nonsuicidal self-injury (NSSI) and suicidal ideation^19^^,^^20^; clinical risk factors, such as hopelessness and depression,^21^ anxiety and agitation,^20^ impulse control difficulties,^19^ and sleep^22^; and interpersonal factors, such as peer victimization^23^ and familial, peer, and school connectedness.^24^^,^^25^ As such, a multifaceted examination of risk and protective factors that span sociodemographic, suicide-related, clinical, and interpersonal domains is needed to better understand predictors of prospective suicide risk in Black youth.

Although predictors of a suicide attempt within 3 months of an ED visit have been reported for the racially and ethnically diverse sample of adolescents in the multisite Emergency Department Screening for Teens at Risk for Suicide (ED-STARS) study,^26^ such predictors have not been reported for Black youth specifically. The primary goal of the present study was to identify risk and protective factors for suicide attempts among Black adolescents (12-17 years of age when recruited) who present to pediatric EDs. The specific aims were 2-fold: to identify sociodemographic and clinical characteristics associated with lifetime suicide attempt at baseline and to determine the predictors of a suicide attempt within 6 months of an adolescent’s index ED visit. To accomplish these aims, we conducted secondary analyses of data from the prospective ED-STARS study, which was conducted in collaboration with the Pediatric Emergency Care Applied Research Network (PECARN).

## Method

### Participants

Study recruitment took place at 13 EDs (PECARN) from June 2015 to July 2016. Study exclusion criteria were as follows: not within the age range, previous study enrollment, ward of state, non–English-speaking adolescents (youth with non–English-speaking parents were enrolled), medically unstable, and severe cognitive impairment. The study team approached 10,664 adolescents (ages 12-17); 6,448 (60.5%) of these adolescents completed a baseline survey assessing sociodemographic variables and suicide risk. Next, 2,897 (43.6%) participants were randomly selected for a 3-month phone interview follow-up, which 2,104 participants completed (72% retention). Participants who completed the follow-up phone interview comprised subgroups of adolescent–parent dyads (*n* = 1,799; 85.5%), adolescents only (*n* = 183, 8.7%), and parents only (*n* = 122, 5.8%).

For this article, the subsample of the larger study that identified as Black (*N* = 1,719) was included in the analyses. Participants were included if they or their parents/guardians indicated their racial identity was Black only (*n* = 1,466) or Black and 1 or more additional races (*n* = 253). The study sample comprised 1,046 girls (61%) and 669 boys (39%) with a mean (SD) age of 15 (1.7) years. Regarding minoritized gender identity status, 12 participants (0.7%) identified as trans male/trans boy, 3 (0.2%) identified as trans female/trans girl, 17 (1.0%) identified as genderqueer/gender nonconforming, and 7 (0.4%) indicated their gender identity was not provided in the list of options presented. Due to small cell sizes, an aggregate genderqueer/gender nonconforming group was created (*n* = 39, 2.3%). A total of 784 participants (45.6% of the Black participants) were selected for follow-up. Additional demographic information is provided in Table 1. For full study procedures, see King *et al.*^26^

**Table 1:** Univariate Relations Between Baseline Variables and Baseline Lifetime Suicide Attempt^a^

	Lifetime suicide attempt/death				*p*^b^	Unadjusted, weighted odds ratio^c^
	Yes (n = 267)		No (n = 1,439)			
Sociodemographic factors						
	**Mean**	**SD**	**Mean**	**SD**		
Age, years	15.24	1.53	14.94	1.67	**.007**	1.12 (1.03, 1.21)
	**n**	**%**	**n**	**%**		
Ethnicity						
Latinx	22	9.7	98	8.30	.503	1.18 (0.71, 1.88)
Not Latinx	205	90.3	1,078	91.7	—	Reference
Gender identity						
Female	194	73.8	772	54.6	—	Reference
Male	50	19.0	624	44.1	**<.001**	0.32 (0.23, 0.44)
Gender minority	19	7.2	18	1.3	**<.001**	4.20 (2.16, 8.21)
Sexual minority						
Straight	139	52.5	1,213	85.3	—	Reference
Bisexual	64	24.2	81	5.7	**<.001**	6.90 (4.75, 10.00)
Mostly straight	28	10.6	62	4.4	**<.001**	3.94 (2.41, 6.31)
Queer	17	6.4	39	2.7	**<.001**	3.80 (2.05, 6.80)
Gay/lesbian	17	6.4	27	1.9	**<.001**	5.49 (2.87, 10.25)
Mother/stepmother education						
No school	N/A		6	0.43	.972	—
High school graduate or less	88	33.8	436	31.3	.249	1.21 (0.87, 1.68)
Some college/technical training	88	33.8	448	32.1	.322	1.18 (0.85, 1.63)
College graduate/professional	84	32.3	504	36.2	—	Reference
Father/stepfather education						
No school	1	0.5	7	0.6	.752	0.71 (0.04, 4.05)
High school graduate or less	128	57.9	638	51.5	—	Reference
Some college/technical training	50	22.6	308	24.9	.241	0.81 (0.56, 1.15)
College graduate/professional	42	19.0	285	23.0	.107	0.73 (0.50, 1.06)
Family public assistance						
Yes	151	56.8	800	56.3	—	Reference
No	115	43.2	620	43.7	.897	0.98 (0.75, 1.28)
Suicidal-related factors						
Suicidal ideation—past week: ASQ question 3						
Yes	99	42.9	43	3.1	**<.001**	23.09 (15.58, 34.75)
No	132	57.1	1,324	96.9		Reference
	**Mean**	**SD**	**Mean**	**SD**		
Suicidal ideation severity—lifetime: C-SSRS	3.84	1.50	0.51	1.15	**<.001**	3.07 (2.76, 3.43)
No. NSSI methods (from Lloyd-Richardson *et al.*)	1.33	1.51	0.13	0.56	**<.001**	3.13 (2.68, 3.69)
	**n**	**%**	**n**	**%**		
NSSI incidents (YRBS)						
0 times	102	38.5	1,319	91.9	—	Reference
1-2 times	68	25.7	88	6.1	**<.001**	9.99 (6.86, 14.55)
3-4 times	38	14.3	14	1.0	**<.001**	35.10 (18.83, 69.04)
5 or more times	57	21.5	15	1.0	**<.001**	49.14 (27.56, 92.83)
Suicidal thoughts—duration: C-SSRS						
Never had suicidal thoughts	19	7.2	1,121	79.8	—	Reference
A few seconds or minutes	45	17.0	129	9.2	**<.001**	20.58 (11.86, 37.03)
Less than 1 h/some of the time	64	24.2	100	7.1	**<.001**	37.76 (22.17, 67.13)
1-4 h/a lot of time	56	21.2	29	2.1	**<.001**	113.93 (61.44, 220.83)
4-8 h/most of day	43	16.3	16	1.1	**<.001**	158.56 (78.28, 339.88)
More than 8 h/continuous	37	14.0	10	0.7	**<.001**	218.30 (98.59, 526.90)
How likely … act on suicidal thoughts (from BSRS)						
No suicidal thoughts/not at all likely	71	27.0	1,332	94.1	—	Reference
Slightly possible	102	38.8	71	5.0	**<.001**	26.95 (18.41, 39.85)
Somewhat likely	55	20.9	10	0.7	**<.001**	103.18 (52.62, 222.81)
Likely	23	8.7	1	0.1	**<.001**	431.49 (89.00, 7,775.25)
Almost for sure will act on them	12	4.6	1	0.1	**<.001**	225.13 (43.46, 4,130.18)
	**Mean**	**SD**	**Mean**	**SD**		
Suicidal rumination (BSRS)	5.79	2.86	0.84	1.88	**<.001**	1.83 (1.73, 1.95)
Clinical and interpersonal risk and protective factors						
	**n**	**%**	**n**	**%**		
Hopelessness item: MFQ						
Not true	100	37.7	1,147	80.2	—	Reference
Sometimes	106	40.0	255	17.8	**<.001**	4.77 (3.52, 6.48)
True	59	22.3	28	2.0	**<.001**	24.17 (14.89, 40.11)
	**Mean**	**SD**	**Mean**	**SD**		
Depression: PHQ-9	13.36	7.64	4.65	5.24	**<.001**	1.20 (1.17, 1.22)
Alcohol use: AUDIT-C score	0.57	1.52	0.11	0.52	**<.001**	1.72 (1.48, 2.02)
	**n**	**%**	**n**	**%**		
Cannabis use: DUS (adapted)						
Never	187	70.8	1,244	87.2	—	Reference
Once or twice	30	11.4	109	7.6	**.006**	1.83 (1.17, 2.79)
Monthly or more	47	17.8	74	5.2	**<.001**	4.23 (2.83, 6.26)
Homicidal thoughts	45	17	78	5.4	**<.001**	3.58 (2.40, 5.28)
	**Mean**	**SD**	**Mean**	**SD**		
Agitation: BAM;	9.82	5.5	3.53	4.81	**<.001**	1.22 (1.19, 1.25)
Anxiety: SCARED-C	3.65	2.44	1.91	1.81	**<.001**	1.44 (1.36, 1.53)
Sleep disturbance: PROMIS	7.08	1.92	6.98	1.88	.436	1.03 (0.96, 1.10)
	**n**	**%**	**n**	**%**		
Physical abuse—family (CTQ-SF)	68	25.7	140	9.8	**<.001**	3.17 (2.28, 4.38)
Sexual abuse (CTQ-SF)	84	31.7	95	6.7	**<.001**	6.48 (4.65, 9.03)
	**Mean**	**SD**	**Mean**	**SD**		
Negative life events	0.96	0.94	0.60	0.76	**<.001**	1.64 (1.41, 1.90)
	**n**	**%**	**n**	**%**		
Functional impairment (PHQ-10)						
Not difficult at all	94	35.5	983	68.5	—	Reference
Somewhat difficult	110	41.5	377	26.3	**<.001**	3.05 (2.26, 4.12)
Very difficult	34	12.8	56	3.9	**<.001**	6.35 (3.92, 10.18)
Extremely difficult	27	10.2	19	1.3	**<.001**	14.86 (8.01, 28.10)
	**Mean**	**SD**	**Mean**	**SD**		
Family connectedness (Parent–Family Connectedness Scale)	6.36	1.97	8.34	1.65	**<.001**	0.58 (0.54, 0.63)
Social/peer connectedness scale (Hemingway Adolescent Connectedness Scale)	6.99	2.39	7.76	1.91	**<.001**	0.84 (0.79, 0.89)
School Connectedness Scale	6.12	2.06	7.41	1.88	**<.001**	0.74 (0.69, 0.78)
Impulsive Aggression Quick Screen (from IPAS)	0.8	1.06	0.29	0.71	**<.001**	1.86 (1.62, 2.13)
Impulsivity: UPPS-P Negative Urgency Subscale	11.1	3.18	8.4	3.58	**<.001**	1.23 (1.19, 1.28)
Peer victimization	3.33	1.87	2.55	1.19	**<.001**	1.38 (1.28, 1.50)
Peer bullying perpetration	2.53	1.31	2.22	0.71	**<.001**	1.39 (1.23, 1.58)
	**n**	**%**	**n**	**%**		
Physical fighting (YRBS)						
0 times	142	53.6	955	66.6	—	Reference
1 time	45	17.0	237	16.5	.188	1.28 (0.88, 1.82)
2 or 3 times	41	15.5	156	10.9	**.004**	1.77 (1.19, 2.58)
4 or more times	37	14.0	85	5.9	**<.001**	2.93 (1.90, 4.45)

Note: ASQ = Ask Suicide-Screening Questions; AUDIT-C = Alcohol Use Disorders Identification Test; BAM = Brief Agitation Measure; BSRS = Brief Suicidal Rumination Scale; C-SSRS = Columbia-Suicide Severity Rating Scale; CTQ-SF = Childhood Trauma Questionnaire, Short Form; DUS = Drug Use Scale; IPAS = Impulsive Premeditated Aggression Scale; MFQ = Mood and Feelings Questionnaire; N/A = not applicable; NSSI = nonsuicidal self-injury; PHQ = Patient Health Questionnaire; PROMIS = Patient-Reported Outcomes Measurement Information System; SCARED-C = Screen for Child Anxiety Related Emotional Disorders child report; UPPS-P = Urgency-Premeditation-Perseverance-Sensation Seeking-Positive Urgency Impulsive Behavior Scale; YRBS = Youth Risk Behavior Surveillance System.

a Missing participant data vary across measures, with a range from 0.12% (age) to 17.90% (ethnicity) missingness.

b All *p* values from univariable logistic regression models. Boldface indicates significant results.

c Upper and lower limits based on 95% CIs for calculated odds ratios are in parentheses.

### Procedures

Procedures were approved by the institutional review board at each site. The 13 pediatric EDs were PECARN members and spanned diverse geographic regions of the United States. At PECARN sites, adolescents were recruited during screening shifts that were randomly selected based on availability of study coordinators and during times (eg, evenings) when there was a higher volume of adolescent patients. Written informed consent and assent were obtained from parents (legal guardians) and adolescents, respectively. Adolescents who turned 18 before follow-up provided consent at that time. The adolescents received a $15 gift certificate for participation.

### Measures

Sociodemographic information about youth was collected from the adolescent and/or the parent or legal guardian and included self-reported sex assigned at birth, race, ethnicity, parental education level, and receipt of public assistance. Adolescent participants completed a survey using a computer tablet, answering questions regarding the following factors:

1. Sociodemographic factors including gender identity and sexual identity
2. Suicide-related risk factors including suicidal behavior (defined using the Columbia Suicide Severity Rating Scale [C-SSRS] to include attempts, aborted, interrupted, and planning behavior) and NSSI (number of incidents and number of different methods used)
3. Clinical risk factors including depression, anxiety, and prior drug and alcohol use
4. Interpersonal factors including past fighting; bullying and victimization; and connectedness to family, friends, and school.

All collected variables, their definitions, and their citations are included in Supplement 1, available online.

We defined 2 primary outcomes, lifetime suicide attempt and follow-up suicide attempt obtained at 3- and/or 6-month follow-up. Lifetime suicide attempt was determined using the C-SSRS. A lifetime suicide attempt was defined by an affirmative answer to either of the following questions: “Have you ever in your life made a suicide attempt?” or “Have you ever in your life tried to harm yourself because you were at least partly trying to end your life?” A follow-up suicide attempt was determined either from parent-reported reason for ED visit/hospitalization at 3- and/or 6-month follow-up (if reported suicide attempt, coded as 1; if noted another reason, coded as 0) or from youth self-reported follow-up surveys using the above-mentioned C-SSRS questions.

### Data Analysis Plan

All analyses were conducted in R version 4.4.1.^27^ Simple logistic regression analyses were used to determine cross-sectional, univariable relations, with lifetime suicide attempt status (yes or no) as the outcome variable and baseline sociodemographic variables, variables pertaining to suicidal thoughts and behaviors and NSSI, and clinical and interpersonal risk and protective factors as predictor variables. Then, prospective, univariable relations, between baseline sociodemographic variables, variables pertaining to suicidal thoughts and behaviors and NSSI, and clinical and interpersonal risk and protective factors and occurrence of a suicide attempt within 6 months of ED visit, were conducted similarly. Variables that approached significance (*p* < .1) in prospective univariable models were candidates for inclusion in a multivariable logistic regression model.^28^

In the first stage of multivariable model selection, sociodemographic variables and variables pertaining to suicidal thoughts and behaviors and NSSI were included in the model using stepwise selection. Variables were either inserted or removed from the model based on Akaike information criterion (AIC) values. The best-fitting model was determined based on achieving lower AIC values. In the second stage, other candidate variables (ie, clinical and interpersonal risk and protective factors) and variables that emerged in the stepwise model were included in a multivariable model using forward selection. Variables were included in the model based on which reduced the AIC value the most until variable inclusion no longer impacted model fit. In the third and final stage, the model selected via forward selection was examined using the backward elimination approach, which arrived at the final model via removal of variables systematically based on reduced AIC values to arrive at the best-fitting model. For the final model, we calculated the area under the curve (AUC) to assess predictive performance of the model, with a 95% CI using the pROC package in R.^29^ We also conducted a 10-fold cross-validation of the model using the caret package in R.^30^

## Results

### Sample Characteristics: Suicidal Thoughts, Suicidal Behaviors, and NSSI

#### Baseline Sample

Of participants included in the baseline sample, 552 (32%) adolescents reported lifetime suicidal ideation, of whom 264 (48%) reported suicidal ideation in the past month, and 281 (16%) reported engaging in NSSI at least once in the past 12 months. In terms of lifetime suicidal behaviors, 1,294 (77%) adolescents reported none; 119 (7%) reported preparatory, interrupted, or aborted attempts; 32 (2%) reported 1 lifetime suicide attempt; and 225 (13%) reported more than 1 lifetime suicide attempt. Responses for suicidal behaviors were missing for 41 adolescents. Of participants who reported at least 1 lifetime suicide attempt, the mean (SD) [median] number of attempts reported was 4 (8.4) [2].

#### Retention: Follow-Up Sample

Of 1,719 Black youths and caregivers who participated in the baseline assessment, a random subset of 784 (45.6%) participants enriched for suicide risk were invited to complete a follow-up assessment at 3- and 6-month follow-up intervals. Briefly, adolescents in the original sample were divided into 3 groups according to baseline data: high risk, including adolescents with suicidal thoughts with intent/plan, history of suicide attempt, NSSI engagement 5 or more times in the past year, or homicidal ideation with intent/plan; moderate risk, including adolescents with suicidal thoughts, homicidal thoughts without intent/plan, or 2 or more suicide risk factors (eg, hopelessness, prior physical or sexual abuse); and low risk, including adolescents not meeting criteria for high- or moderate-risk group. In the follow-up interval, 83.6% of high-risk youth, 68% of moderate-risk youth, and 16.3% of low-risk youth were randomized to follow-up. Additional details can be found in King *et al.*^26^ Of these, 616 youths provided data on suicide attempt status (78.6% retention rate) at 3- and/or 6-month follow-up, of which 38 (6%) youths reported a suicide attempt between their ED visit and 3- and/or 6-month follow-up. No differences in terms of ethnicity (Hispanic vs non-Hispanic), family public assistance, mother’s education level, gender identity, or sexual minority status emerged between participants who completed the follow-up and participants who were invited to, but did not, complete the follow-up. Significant differences between participants who completed the follow-up and those who did not based on father’s education level (χ^2^(3) = 8.38, *p* = .039) emerged, with higher education levels seen for participants who completed the follow-up.

### Predictors of Suicide Attempt Univariable Findings

#### Cross-Sectional Associations

Gender identity; sexual minority status; age; and all suicidal ideation and behavior, NSSI, and other risk and protective factors except sleep were significantly related to lifetime suicide attempt status (Table 1).

#### Prospective Associations

Gender identity, sexual minority status, and all suicidal ideation and behavior and NSSI factors predicted follow-up suicide attempt status. Regarding other clinical and interpersonal risk and protective factors, hopelessness, depression, agitation, anxiety, sexual abuse history, level of impairment (Patient Health Questionnaire-10), all forms of connectedness (family, peer, school), and impulsivity predicted follow-up suicide attempt status (Table 2).

**Table 2:** Univariate Relations Between Baseline Variables and Follow-Up Suicide Attempt at 3- or 6-Month Time Point^a^

	Follow-up suicide attempt/death				*p*^b^	Unadjusted, weighted odds ratio^c^
	Yes (*n* = 39)		No (*n* = 577)			
Sociodemographic factors						
	**Mean**	**SD**	**Mean**	**SD**		
Age, y	15.24	1.51	15.13	1.61	.666	1.05 (0.85, 1.29)
	**n**	**%**	**n**	**%**		
Ethnicity						
Latinx	2	5.7	42	8.5	.567	0.65 (0.10, 2.25)
Not Latinx	33	94.3	452	91.5	—	Reference
Gender identity						
Female	30	76.9	351	61.7	—	Reference
Male	5	12.8	203	35.7	**.011**	0.29 (0.10, 0.69)
Gender minority	4	10.3	15	2.6	**.055**	3.12 (0.85, 9.25)
Sexual minority						
Straight	17	43.6	409	71.3	—	Reference
Bisexual	14	35.9	68	11.8	**<.001**	4.95 (2.30, 10.51)
Mostly straight	4	10.3	36	6.3	.091	2.67 (0.74, 7.69)
Queer	2	5.1	33	5.7	.624	1.46 (0.23, 5.39)
Gay/lesbian	2	5.1	28	4.9	.483	1.72 (0.26, 6.41)
Mother/stepmother education						
No school	N/A		3	0.5	.988	—
High school graduate or less	16	41.0	173	30.6	.429	1.36 (0.64, 2.95)
Some college/technical training	10	25.6	199	35.2	.483	0.74 (0.31, 1.72)
College graduate/professional	13	33.3	191	33.7	—	Reference
Father/stepfather education						
No school	N/A		3	0.6	.988	—
High school graduate or less	13	37.1	257	52.4	—	Reference
Some college/technical training	12	34.3	128	26.1	.137	1.85 (0.81, 4.20)
College graduate/professional	10	28.6	102	20.8	.130	1.94 (0.80, 4.55)
Family public assistance						
Yes	23	59.0	334	58.3	—	Reference
No	16	41.0	239	41.7	.933	0.97 (0.49, 1.87)
Suicidal-related factors						
Suicidal ideation—past week: ASQ question 3						
Yes	21	61.8	67	12.7	**<.001**	11.14 (5.39, 23.85)
No	13	38.2	462	87.3	—	Reference
	**Mean**	**SD**	**Mean**	**SD**		
Suicidal ideation severity—lifetime: C-SSRS	3.85	1.53	1.67	1.90	**<.001**	1.84 (1.52, 2.31)
No. NSSI methods (from Lloyd-Richarson et al.)	1.56	1.45	0.46	1.00	**<.001**	1.79 (1.45, 2.20)
	**n**	**%**	**n**	**%**		
NSSI incidents (from YRBS)						
0 times	9	23.1	432	75.0	—	Reference
1-2 times	16	41.0	83	14.4	**<.001**	9.25 (4.03, 22.53)
3-4 times	5	12.8	22	3.8	**<.001**	10.91 (3.14, 34.47)
5 or more times	9	23.1	39	6.8	**<.001**	11.08 (4.10, 29.99)
Suicide attempts/behavior—lifetime						
None	3	7.7	358	63.3	—	Reference
Aborted/interrupted attempt only	3	7.7	79	14.0	**.067**	4.53 (0.83, 24.88)
One previous suicide attempt	1	2.6	20	3.5	.129	5.97 (0.29, 49.03)
Multiple previous attempts	32	82.1	109	19.3	**<.001**	35.03 (12.24, 147.78)
Suicidal thoughts—duration: C-SSRS						
Never had suicidal thoughts	3	7.9	274	48.6	—	Reference
A few seconds or minutes	2	5.3	98	17.4	.492	1.86 (0.24, 11.41)
Less than 1 h/some of the time	6	15.8	99	17.6	**.040**	5.54 (1.43, 26.62)
1-4 h/a lot of time	12	31.6	40	7.1	**<.001**	27.40 (8.29, 124.13)
4-8 h/most of day	10	26.3	27	4.8	**<.001**	33.83 (9.69, 157.83)
More than 8 h/continuous	5	13.2	26	4.6	**<.001**	17.56 (4.08, 89.63)
How likely … act on suicidal thoughts (from BSRS)						
No suicidal thoughts/not at all likely	9	23.7	426	75.0	—	Reference
Slightly possible	10	26.3	95	16.7	**.001**	4.98 (1.96, 12.87)
Somewhat likely	11	28.9	31	5.5	**<.001**	16.80 (6.49, 44.70)
Likely	7	18.4	13	2.3	**<.001**	25.49 (8.05, 79.84)
Almost for sure will act on them	1	2.6	3	0.5	**.022**	15.78 (0.74, 137.86)
	**Mean**	**SD**	**Mean**	**SD**		
Suicidal rumination (BSRS)	6.61	2.88	2.56	3.02	**<.001**	1.45 (1.31, 1.64)
Clinical and interpersonal risk and protective factors						
	**n**	**%**	**n**	**%**		
Hopelessness item: MFQ						
Not true	10	25.6	364	63.1	—	Reference
Sometimes	15	38.5	173	30.0	**.006**	3.16 (1.40, 7.39)
True	14	35.9	40	6.9	**<.001**	12.74 (5.36, 31.39)
	**Mean**	**SD**	**Mean**	**SD**		
Depression: PHQ-9	15.70	8.16	7.95	6.73	**<.001**	1.14 (1.09, 1.19)
Alcohol use: AUDIT-C score	0.21	0.57	0.27	1.03	.681	0.92 (0.54, 1.24)
	**n**	**%**	**n**	**%**		
Cannabis use: DUS (adapted)						
Never	32	82.1	449	78.2	—	Reference
Once or twice	4	10.3	68	11.8	.725	0.83 (0.24, 2.16)
Monthly or more	3	7.7	57	9.9	.625	0.74 (0.17, 2.15)
Homicidal thoughts	4	10.3)	54	9.4	.853	1.11 (0.32, 2.90)
	**Mean**	**SD**	**Mean**	**SD**		
Agitation: BAM	11.10	5.31	6.20	5.61	**<.001**	1.16 (1.10, 1.24)
Anxiety: SCARED-C	4.51	2.57	2.59	2.10	**<.001**	1.39 (1.22, 1.59)
Sleep disturbance: PROMIS	7.28	1.57	7.07	1.96	.511	1.06 (0.89, 1.24)
	**n**	**%**	**n**	**%**		
Physical abuse—family (CTQ-SF)	8	20.5	117	20.5	.993	1.00 (0.42, 2.14)
Sexual abuse (CTQ-SF)	12	30.8	99	17.4	**.040**	2.11 (1.00, 4.23)
	**Mean**	**SD**	**Mean**	**SD**		
Negative life events	0.68	0.62	0.8	0.83	.386	0.83 (0.52, 1.25)
	**n**	**%**	**n**	**%**		
Functional impairment (PHQ-10)						
Not difficult at all	9	23.1	299	51.9	—	Reference
Somewhat difficult	20	51.3	206	35.8	**.004**	3.23 (1.48, 7.58)
Very difficult	6	15.4	51	8.9	**.013**	3.91 (1.26, 11.31)
Extremely difficult	4	10.3	20	3.5	**.003**	6.64 (1.69, 22.42)
	**Mean**	**SD**	**Mean**	**SD**		
Family connectedness (Parent–Family Connectedness Scale)	6.54	1.68	7.51	1.89	**.002**	0.78 (0.66, 0.91)
Social/peer connectedness scale (Hemingway Adolescent Connectedness Scale)	6.10	2.57	7.49	2.13	**<.001**	0.77 (0.67, 0.88)
School Connectedness Scale	5.28	2.18	6.85	2.02	**<.001**	0.72 (0.62, 0.83)
Impulsive Aggression Quick screen (from IPAS)	0.72	0.92	0.53	0.91	.217	1.22 (0.87, 1.66)
Impulsivity: UPPS-P Negative Urgency Subscale	11.32	3.25	9.84	3.47	**.012**	1.14 (1.03, 1.26)
Peer victimization	3.28	1.73	2.94	1.60	.207	1.12 (0.93, 1.32)
Peer bullying perpetration	2.50	1.13	2.35	0.96	.368	1.13 (0.82, 1.45)
	**n**	**%**	**n**	**%**		
Physical fighting (YRBS)						
0 times	21	55.3	343	59.4	—	Reference
1 time	7	18.4	100	17.3	.766	1.14 (0.44, 2.65)
2 or 3 times	6	15.8	81	14.0	.691	1.21 (0.43, 2.93)
4 or more times	4	10.5	53	9.2	.711	1.23 (0.35, 3.39)

Note: ASQ = Ask Suicide-Screening Questions; AUDIT-C = Alcohol Use Disorders Identification Test; BAM = Brief Agitation Measure; BSRS = Brief Suicidal Rumination Scale; C-SSRS = Columbia-Suicide Severity Rating Scale; CTQ-SF = Childhood Trauma Questionnaire, Short Form; DUS = Drug Use Scale; IPAS = Impulsive Premeditated Aggression Scale; MFQ = Mood and Feelings Questionnaire; N/A = not applicable; NSSI = nonsuicidal self-injury; PHQ = Patient Health Questionnaire; PROMIS = Patient-Reported Outcomes Measurement Information System; SCARED-C = Screen for Child Anxiety Related Emotional Disorders child report; UPPS-P = Urgency-Premeditation-Perseverance-Sensation Seeking-Positive Urgency Impulsive Behavior Scale; YRBS = Youth Risk Behavior Surveillance System.

a Missing participant data vary across measures, with a range from 0.16% (physical fighting, functional impairment, NSSI incidents, suicidal ideation severity—lifetime) to 14.80% (father/stepfather education) missingness.

b All *p* values from univariable logistic regression models. Boldface *p* values indicate significant results.

c Upper and lower limits based on 95% CIs for calculated odds ratios are in parentheses.

### Multivariable Prospective Model

The final multivariable model, based on stepwise, forward, and backward selection (see “Data Analysis Plan”) included the following variables: suicidal behavior, suicidal ideation in the past week at baseline, family connectedness, school connectedness, peer connectedness, and impulsivity (AUC = 0.91, 95% CI 0.87-0.95) (Table 3). In the 10-fold cross-validation analyses, the median (interquartile range) AUC was 0.92 (0.87-0.94).

**Table 3:** Multivariable Model Predicting Follow-Up Suicide Attempt Status

Baseline characteristics	Suicide attempt at 3-6 months				OR (95% CI)	*p*
	Yes (*n* = 33)		No (*n* = 506)			
	**n**	**%**	**n**	**%**		
Lifetime suicidal behavior (reference: none)				-		
Preparatory, aborted, or interrupted	2	6.1	66	13.0	2.35 (0.29, 15.46)	.373
One suicide attempt	1	3.0	18	3.6	5.31 (0.23, 51.84)	.187
Multiple suicide attempts	27	81.8	92	18.2	23.09 (6.54, 110.06)	.000
Past-week suicidal ideation (reference: no)				-		
Yes	21	63.6	63	12.5	3.96 (1.58, 10.38)	.004
	**Mean**	**SD**	**Mean**	**SD**		
Family connectedness	6.79	1.67	7.57	1.88	1.45 (1.12, 1.91)	.007
School connectedness	5.36	2.16	6.95	1.99	0.83 (0.65, 1.05)	.122
Social connectedness	6.09	2.69	7.57	2.10	0.86 (0.70, 1.04)	.129
Impulsivity	11.48	3.20	9.81	3.50	1.13 (0.99, 1.30)	.090

Note: Candidate variables based on *p* < .1 in univariable prospective logistic regression models included the following: gender identity, sexual minority, nonsuicidal self-injury (number of incidents and number of methods), lifetime suicide behavior/attempt status (C-SSRS), suicidal ideation duration (C-SSRS), likelihood of acting on suicidal thoughts (BSRS), suicidal rumination (BSRS), past-week suicidal ideation (Ask Suicide-Screening Questions), lifetime suicidal ideation severity (C-SSRS), hopelessness (Mood and Feelings Questionnaire), depression (PHQ-9), agitation (Brief Agitation Measure), anxiety (Screen for Child Anxiety Related Emotional Disorders), sexual abuse (Childhood Trauma Questionnaire, Short Form), level of impairment (PHQ-10), family connectedness, social/peer connectedness, school connectedness, impulsivity (Urgency-Premeditation-Perseverance-Sensation Seeking-Positive Urgency Impulsive Behavior Scale Negative Urgency). Missing data were removed using listwise deletion (13%-17% missingness). BSRS = Brief Suicidal Rumination Scale; C-SSRS = Columbia-Suicide Severity Rating Scale; OR = odds ratio; PHQ = Patient Health Questionnaire.

A closer look at the multivariable model revealed that for suicidal behavior, only multiple attempt status was a significant predictor of follow-up suicide attempt status compared with no suicidal behavior engagement, whereas other suicidal behaviors, such as aborted and interrupted attempts, did not significantly differentiate groups. Endorsement of suicidal ideation in the past week at baseline was also a significant predictor of follow-up attempt status. Family connectedness positively predicted suicide attempt status at follow-up (*B* = .37, SE = 0.14, *p* = .007), whereas school connectedness, peer connectedness, and impulsivity were nonsignificant predictors (all *p* > .090).

## Discussion

This study is among the first studies to examine cross-sectional and prospective risk for suicide attempts among a large cohort of Black adolescents who presented to a sample of nationally representative and geographically varied pediatric EDs. We identified sociodemographic, suicide-related, clinical, and interpersonal characteristics of youth associated with lifetime suicide attempt and prospectively identified predictors of a suicide attempt within 6 months of ED visits. There was substantial convergence between correlates of suicidal attempts at baseline and prospective predictors of suicide attempts within 6 months. Specifically, gender identity, sexual minority status, and all suicidal ideation and behavior and NSSI factors predicted both baseline and follow-up suicide attempt status. Regarding other clinical and interpersonal risk and protective factors, hopelessness, depression, agitation, anxiety, sexual abuse history, functional impairment, impulsivity, and all forms of connectedness (family, peer, school) predicted baseline and prospective suicide attempt status. Of note, the following variables increased the prospective odds of a suicide attempt by 5 or more times: past-week ideation, number of NSSI incidents, prior suicide attempts, suicidal ideation duration lasting less than 1 hour, 1-4 hours, or 4-8 hours in a day, likelihood of acting on suicidal thoughts (somewhat likely or more), hopelessness, and high levels of functional impairment.

Although we found that suicidal ideation and higher rates of depression and anxiety were both cross-sectionally and prospectively related to suicide attempt outcomes, prior work has shown that Black adolescents had greater odds of a suicide attempt without the presence of suicidal ideation,^31^ and, in a national sample, Black adolescents were less likely than White adolescents to have received a diagnosis of a mental health disorder before death by suicide.^12^ Among Black adolescents in the present sample who reported a suicide attempt at follow-up, 33% denied suicidal ideation in the past week at baseline, whereas 54% reported suicidal ideation in the past week at baseline (in line with the overall sample; see King *et al.*^26^). Our findings suggest that several clinical risk factors confer risk for prospective suicide attempts at high levels, including hopelessness and severe functional impairment, which could inform clinical decision making in the absence of suicidal ideation and formal mental health diagnosis. Future work should replicate and extend these findings by identifying additional variables that confer high risk in Black youth in the absence of suicidal thoughts or a mental health diagnosis.

Of note, 13% of Black youth in this sample who did not respond to the baseline question asking about past-week suicidal ideation attempted suicide at the follow-up interval. This is distinct from the overall sample, in which only 8% of participants who attempted suicide at follow-up did not respond to this question. Prior work has illustrated greater levels of nonresponse to suicide-related questions in Black youth,^32^ which may be related to cultural stigma surrounding suicide disclosures, social desirability effects, and reduced comfort with formal assessments.^32^, ^33^, ^34^ Nonresponse to suicide questions may also partially explain why suicidal ideation has been found to be a less potent predictor of follow-up suicide attempts in Black youth.^31^

Additionally, 26% of Black adolescents in this sample did not meet the recommended cutoff score for depression (Patient Health Questionnaire-9), but reported a suicide attempt at follow-up. Given the small sample sizes, we were unable to examine predictive models specific to each of these subgroups; we strongly encourage researchers to focus on identifying different measurement tools and unique risk factors that will increase predictive accuracy of suicide attempts in Black adolescents. One promising approach includes the newly developed Kiddie Computerized Adaptive Test Suicide Scale (K-CAT-SS),^35^ a tool that relies on a large number of items that are adaptively administered based on a patient’s response to prior questions. Such an approach could be helpful in assessing suicide risk in Black youth, given that suicidal thoughts and other risk factors are more likely to be missed in this population.^36^ In another investigation, the Ask Suicide Screening Questions^37^ tool captured an additional 55% of patients who did not report suicidal thoughts and behaviors as their presenting concern on arrival to a pediatric ED, a subsample that was more likely to be male, Black, or Latino compared with White, other race, or Asian, highlighting the importance of multiple screening tools to capture suicide risk in this population.^38^

Although our findings largely converge with previous findings related to suicide risk in adolescence^39^, ^40^, ^41^ and align with findings from the original ED-STARS sample of adolescents characterized by racial and ethnic diversity,^26^ these results extend our current understanding of risk for suicide attempts in Black youth. In particular, our findings highlight the importance of intersectional identities (ie, gender and sexual minority status) and of protective interpersonal factors. Intersectionality theory posits that having multiple marginalized identities (eg, Black and sexual minority and/or gender minority) results in a unique experience at the intersection of both identities, leading to greater levels of discrimination and victimization that are multiplicative and greater than the sum of each identity’s parts.^42^^,^^43^ Preliminary work has exhibited that people with multiple marginalized identities are at higher risk for depression,^44^ whereas findings for suicide attempts have been mixed.^45^, ^46^, ^47^ These findings amplify the need to advance our understanding of the unique experiences of Black youth who also identify as a sexual or gender minority. Beyond identifying disparities in suicide rates, future work should also consider the pathways leading to heightened suicide risk in transgender and gender expansive Black youth, which, based on prior work, could include peer victimization, symptoms of depression, substance use, and dating violence.^48^

In addition, and in line with the heuristic model posited by Robinson *et al.*^49^ for suicidal risk among African American youth and the importance of understanding suicide risk in Black youth via resilience and strength-based perspectives, our findings provide support for the importance of school, family, and peer connectedness in reducing risk for suicide attempts—both at baseline and during follow-up periods. Prior work has found some evidence that different forms of connectedness are related to reductions in suicide risk in Black youth,^2^ stressing the importance of supporting community-led approaches in suicide prevention efforts aimed at Black youth specifically.

Notably, we did not find an association between sleep difficulties and lifetime or prospective suicide risk in this sample. Although such an association has been previously demonstrated^22^ and was found in the original ED-STARS study,^26^ at least 1 investigation has shown that the sleep–suicide risk association was weaker for Black and Hispanic youth.^50^ In their investigation, Joseph *et al.*^50^ note that this could be due to underreporting of mental health difficulties in Black youth, pointing to the importance of developing screening and assessment tools that are culturally responsive. Molock *et al.*^36^ note that, whereas some assessment tools have been validated in Black youth samples, whether they function equally across populations is seldom investigated. As such, tools developed specifically for Black youth that consider novel risk and protective factors that are relevant to this population are needed. Molock *et al.*^36^ recommend that such an assessment include societal context (eg, stigma, discrimination, acculturation), institutional context (eg, availability of resources), neighborhood context (eg, safe outdoor spaces, exposure to violence), and familial context (eg, intergenerational trauma). Future work should aim to identify novel risk and protective factors, integrate these with known risk factors, and develop tools in collaboration with Black youth community partners.

Findings from the multivariable analysis also partly converged with previous work, with multiple suicide attempt status and past-week suicidal ideation (measured at baseline) predicting greater likelihood of a suicide attempt at 3- and 6-month follow-up. Given that suicide-related indicators are among the most potent predictors of follow-up suicide attempts, clinicians should take these findings into account when supporting youth in the ED (eg, administering assessments, safety planning), while also communicating about suicide in a culturally sensitive manner. Given prior work illustrating health care disparities in medical settings,^51^ Hartford *et al.*^52^ developed a framework for improving patient equity in pediatric EDs (eg, development of ED equity committee workshops on racism, bias, and microaggressions). Although not yet formally tested, such a framework aims to improve ED care for Black youth and, given its promise, needs future investigation. Beyond the ED, findings also highlight the importance of decreasing stigma and fostering conversations about suicide disclosures in Black communities to strengthen early detection of risk and mitigate future risk.

Surprisingly, family connectedness was positively related to suicide attempt status in the multivariable model. Importantly, however, mean levels of family connectedness were lower in the suicide attempt group compared with the non–suicide attempt group. As such, it is likely that family connectedness and other variables in our model interacted with one another in meaningful ways, suggesting that family connectedness is protective in some cases, but not in others. For instance, this finding may relate to the intersection of sexual or gender minority identity of Black youth and family dynamics, resulting in fears of coming out due to a perceived or actual rejection within their family or broader Black community, despite feeling a high sense of family connectedness—highlighting a potential interaction between sexual or gender minority status and family connectedness. Whereas most previous studies have found connectedness to be a protective factor in high-risk youth,^53^ there are notable exceptions. In 1 study, the connection between parental caring and lower suicidal ideation was stronger in girls than in boys,^54^ and in a systematic review, familial variables were found to be less associated with suicide risk in urban and low-income Black youth.^55^ It is unclear at this point why family connectedness was positively related to suicide attempt status in our multivariable model; future research is expected to closely examine the nuanced association between each of the connectedness factors and suicide attempt status in Black youth in considering their possible multiplicative effects and should assess potential moderators of this association.

It is important to note that our univariable models consistently showed the protective role of family connectedness, highlighting the importance of developing family-based interventions to support high-risk youth. Whereas a prior review identified 2 well-established and evidenced-based family interventions for youth at risk for suicide,^56^ a recent systematic and meta-analytic analysis identified only 1 intervention, attachment-based family therapy,^57^ that was culturally adapted for suicidal Black youth. Although there are several emerging interventions focused on Black youth suicide (for an overview, see Goodwill^58^), there remains an urgent need to develop and establish suicide interventions for Black youth specifically.^59^

This study had multiple strengths, including its relatively large sample of Black adolescents recruited from 13 pediatric EDs that were characterized by geographic diversity, the broad range of suicide risk factors assessed, and the study’s prospective design. Nevertheless, this study also had several important limitations. First, our categorization of racial identity did not capture the diversity of Black racial identities (eg, Cuban, Dominican, Haitian, Jamaican). This would have strengthened the study, particularly given the history of slavery and structural racism for African Americans in the United States^49^ that may uniquely impact risk and protective factors for this subgroup. Relatedly, due to limitations in sample size, an aggregate genderqueer/gender nonconforming group was created, which obscures unique associations between specific gender identities and suicide risk in Black youth.

As a third weakness, the original ED-STARS study assessment battery was designed to develop a suicide risk screen and focused much more heavily on suicide risk rather than resilience. Except for connectedness to school, friends, and family, we are missing information on other protective factors (eg, racial identity, coping strategies, spirituality and religious engagement) and, more broadly, a strengths-based perspective that may be important for informing a pathway to resilience^49^ for Black adolescents.

Further, the original ED-STARS study assessment focused more heavily on identifying predictors of risk that cut across different identities, thereby neglecting important factors that are unique to Black adolescents, including experiences of discrimination and systemic racism, both of which have been theorized and/or shown to increase suicide risk in this population.^60^, ^61^, ^62^ Similarly, the original ED-STARS study was limited in its assessment of social determinants of health, and some of those that were investigated (eg, public assistance) did not emerge as significant predictors of suicide attempt status in univariable models. Future research should consider a broader investigation of social determinants of health that more sensitively captures disparities given prior work illustrating the importance of these factors in predicting suicide risk.^63^

Moreover, this study was conducted primarily in pediatric EDs of academic health systems, which are not representative of the range of medical EDs in the United States. This is especially important in considering prior work from the overall sample illustrating a link between reduced mental health service utilization and racial and ethnic minority youth.^64^ Also, despite our relatively large sample of Black adolescents, the number of Black adolescents who reported a suicide attempt between their ED visit and ED-STARS follow-up interview was relatively small (*n* = 39). This reduced our statistical power for analyses of multiple predictors of suicide attempts at 1 time and their interactions and further highlights the importance of examining univariable predictors of suicide attempts, even if they may overlap in their predicative utility of future suicide attempts.

Finally, this study focused exclusively on risk and protective factors that were related to lifetime or prospective suicide attempt and/or death. As such, an investigation of predictors of prospective suicidal ideation was out of the scope of this study, although this is a worthwhile goal given the immense suffering that typically accompanies suicidal ideation. Future work should focus on predictors of suicidal ideation in Black youth, particularly given prior work highlighting risk factors that may be unique to suicidal thoughts but not behaviors.^19^

This is one of a few large-scale studies that has prospectively investigated risk factors of suicide attempts in Black youth, adding to the limited literature in this area that has primarily focused on suicide death outcomes (using nationally available databases), suicidal ideation, and cross-sectional analyses. Our findings converged with prior work, highlighting the importance of a range of suicide-related risk factors, clinical risk factors, and connectedness. In univariable models and of note, hopelessness and severe functional impairment emerged as strong predictors of prospective suicide attempt. Additionally, sexual and gender minority status, a range of suicidal behaviors, and connectedness across school, peers, and family all predicted follow-up suicide attempt status. In multivariable models, multiple attempt status, suicidal ideation, and family connectedness emerged as important predictors. Future research should build predictive models of suicide risk in Black youth that also consider unique socioecological and contextual factors, risk and protective factors, and intersectional identities to ensure that the nuanced experiences of Black youth living in the United States are captured.

## CRediT authorship contribution statement

**Nadia Al-Dajani:** Writing – review & editing, Writing – original draft, Visualization, Formal analysis, Data curation, Conceptualization. **Amanda Jiang:** Writing – review & editing, Writing – original draft, Visualization. **David A. Brent:** Writing – review & editing, Methodology, Investigation, Funding acquisition. **Jacqueline Grupp-Phelan:** Writing – review & editing, Methodology, Investigation, Funding acquisition. **T. Charles Casper:** Writing – review & editing, Methodology, Conceptualization. **Polly Y. Gipson Allen:** Writing – review & editing, Writing – original draft. **Cheryl A. King:** Writing – review & editing, Writing – original draft, Project administration, Methodology, Investigation, Funding acquisition, Conceptualization.
