# Supplementary material for: Prospective Risk and Protective Factors for Suicide Attempts Among Black Adolescents Seeking Emergency Department Services
Source: JAACAP Open. 2025 Nov 24;4(2):310–24. doi: 10.1016/j.jaacop.2025.11.002 (PMC13043464; doi:10.1016/j.jaacop.2025.11.002)
Supplement: Supplemental Material [file mmc1.docx]

SUPPLEMENT 1

**Measures**

**Sociodemographic Factors**

At baseline, adolescent and parents’ self-reported demographics, including age, gender, sexuality, racial and ethnic identification, grade in school, mother and father’s educational level, and welfare status.

**Suicide-Related Factors**

*Adolescent Suicidal Ideation.* Past week suicidal ideation was assessed with the Ask Suicide-Screening Questions (ASQ) (Horowitz et al., 2012). In this study we used the third item of the ASQ “In the past week, have you been having thoughts about killing yourself?” The ASQ has strong sensitivity, specificity, and negative predictive value among pediatric samples (Horwitz et al., 2020). The ASQ has demonstrated high sensitivity, correctly identifying approximately 96% of youth at risk for suicide among those who gave an affirmative response to at least one ASQ, when compared with the gold standard assessment (Horwitz et al., 2020).

*Adolescent Suicidal Ideation Severity and Attempt*. The Columbia-Suicide Severity Rating Scale (C-SSRS) Suicidal Ideation Severity Scale (Posner et al., 2011) was used to assess suicidal ideation severity. The scale had a range from 0 ‘no suicidal ideation’ to 5 ‘suicidal ideation with specific plan and intent.’ The item assessing the duration of suicidal thoughts from C-SSRS intensity scale was administered at baseline to adolescents with scores of 1 (‘Wish to be dead’) or 2 (‘Non-specific active suicidal thoughts’) on the Suicidal Ideation Severity Scale. Adolescents and parents completed an adapted version of the C-SSRS Behavior Scale, which included items with yes/no responses about lifetime suicide attempts and suicide-related behaviors (i.e., planning, aborted, and interrupted attempts). Planning behaviors were assessed using the following: “Have you ever in your life taken any steps toward making a suicide attempt or preparing to kill yourself? Examples: saving pills, getting a gun, giving your things away, writing a suicide note. Aborted attempts were assessed using the following: Have you ever in your life started to do something to end your life but stopped yourself before you actually did anything? Examples: took out pills but didn't swallow any, held a gun but changed your mind, went to the roof but didn't jump. Finally, interrupted behaviors were assessed using the following: Have you ever in your life started to do something to end your life but someone or something stopped you before you did anything? The C-SSRS has demonstrated strong validity with other multi-informant suicidal ideation and behavior scales and high sensitivity and specificity for suicidal behavior classifications compared with another behavior scale and an independent suicide evaluation board (Cha et al., 2023; Mayes et al., 2023; Posner et al., 2011). The C-SSRS has also been extensively used in adolescent psychiatric samples (Mayes et al., 2023; Posner et al., 2011).

*Adolescent Suicidal Rumination*. Suicidal rumination (“When I have suicidal thoughts, it is hard to think about other things” and “My suicidal thoughts repeat over and over in my head”) was assessed with the Brief Suicidal Rumination Scale. This two-item measure was developed by the lead author for the initial study (King et al., 2019). The internal consistency for the current sample was 0.94.

*Likelihood to act on suicidal thoughts*. Likelihood of acting on suicidal thoughts (“How likely are you to act on your suicidal thoughts?”) was assessed using one item from the Brief Suicidal Rumination Scale developed by the lead author for the initial study (King et al., 2019).

*Adolescent Non-Suicidal Self-Injury (NSSI)*. NSSI was assessed using one item from the Youth Risk Behavior Survey, administered by the U.S. Center for Disease control to assess health-risk behaviors, and includes reliable items assessing NSSI (Brener et al., 2002; Centers for Disease Control and Prevention, 2012): “During the past 12 months, how many times did you do something to purposely hurt yourself without wanting to die, such as cutting or burning yourself on purpose?” If the response was affirmative, then additional questions were asked about which method(s) the participant have used to hurt themselves over the last 12 months (Lloyd et al., 1997; Lloyd-Richardson et al., 2007). Methods derived from a previously validated checklist (Lloyd et al., 1997) and included the following options: (1) cutting or carving on skin, (2) picking at a wound, (3) hitting self, (4) scraping skin to draw blood, (5) biting self, (6) picking areas of the body to the point of drawing blood, (7) inserting objects under skin or nails, (8) tattooing self, burning skin, (9) pulling out one’s own hair, (10), erasing skin to draw blood, and (11) other.

**Clinical and Interpersonal Risk and Protective Factors**

*Adolescent Homicidal Thoughts*. Adolescents’ homicidal thoughts were assessed using a two-item scale developed for the initial study (King et al., 2019). Items included: “In the past month, have you had any thoughts about wanting to kill someone else?” and “If yes, do you currently have any intent or plan to kill someone?”

*Adolescent Depression*. Depression (“feeling down, depressed, or hopeless”) was assessed using the Patient Health Questionnaire (PHQ-9), a nine-item scale asking about the frequency of depressive symptoms over the past two weeks on a 4-point Likert scale (Kroenke et al., 2001). The internal consistency for the current sample was 0.90.

*Adolescent Hopelessness*. Hopelessness was assessed using one item (“I thought there was nothing good for me in the future”) from the 33-item Mood and Feelings Questionnaire (MFQ) on a 3-point Likert scale (Angold et al., 1995). The full MFQ has been validated in clinical and community settings among children and adolescents and has high internal consistency, good discriminative validity, and good test-retest reliability (Burleson Daviss et al., 2006; Kent et al., 1997; Wood et al., 1995).

*Adolescent Anxiety.* Anxiety symptoms (“People tell me that I worry too much.”) were assessed using the Screen for Child Anxiety Related Disorders (SCARED-C) Short Version, a 3-point Likert scale that examines adolescents’ anxious symptoms over the last 3 months (Birmaher et al., 1999). The internal consistency for the current sample was 0.64.

*Adolescent Agitation*. Agitation (“Recently, I feel a lot of emotional turmoil in my gut “) was assessed with The Brief Agitation Measure (BAM), a 3-item self-report that uses a 7-point Likert scale (Ribeiro et al., 2011). The internal consistency for the current sample was 0.87.

*Sleep Quality*. Sleep quality (“My sleep was refreshing”) over the past week was assessed with the Patient-Reported Outcomes Measurement Information System (PROMIS) Sleep – Short Form. The PROMIS is a 4-item self-report measure that uses a 5-point Likert scale (Cella et al., 2007). The internal consistency for the current sample was 0.86

*Adaptive Functioning*. Adaptive functioning was assessed with the modified version of the functional impairment question from Patient Health Questionnaire (PHQ-10) completed by adolescents and parents (Eisenberg et al., 2013; Kroenke et al., 2001). For the current study, the item was modified to ask: “Over the last two weeks, how hard has it been for you to do what you need to do and get along with others?” The global self-assessment of functional impairment associated with a patient’s psychological symptoms (item 10) has been shown to be a potent indicator of the likelihood of a diagnosis of depression (Spitzer et al., 1999).

*Adolescent Alcohol and Drug Use.* Alcohol consumption was assessed with the Alcohol Use Disorders Identification Test-Consumption (AUDIT) (Saunders et al., 1993). The internal consistency for the current sample was 0.80. Frequency of drug use during the past three months was assessed with the National Institute on Drug Abuse – Modified Alcohol, Smoking, Substance Involvement Screening Test (ASSIST version 31) of the Drug Use Scale (DUS) (Nock et al., 2010). The ASSIST has demonstrated strong reliability and validity and can detect unhealthy substance use (Gryczynski et al., 2015; McNeely et al., 2014). In the study, we used a binary variable to indicate whether or not the adolescent had used cannabis at least monthly in the past three months.

*Adolescent Impulsive Aggression and Impulsivity.* Adolescent impulsive aggression was measured with the Impulsive-Aggression (IA) Quick Screen, which included a one-item screen from the Impulsive Premeditated Aggression Scale (IPAS) (Stanford et al., 1995). The question is as follows: “Over the past three months, have you had times when you became angry and enraged with others in a way that was out-of-control or inappropriate?” If the participant answered “yes”, then they were asked if they have done any of the following during these times: verbally attacked someone, throw or destroyed objects, physically attacked someone. The score is the number of these items endorsed. The internal consistency for the current sample was 0.74. To assess impulsivity (“When I am upset I often act without thinking”), the adolescents completed an adapted version of the Urgency-Premeditation-Perseverance-Sensation Seeking-Positive Urgency Negative Urgency Subscale, a four-item subscale that uses a 4-point Likert scale to assess impulsivity (Dugré et al., 2019; Miller et al., 2012). We revised the wording of 3 out of 4 previously validated items from the four-item subscale to make them more understandable for our adolescent sample (Dugré et al., 2019). For example, the original item “When I feel rejected, I will often say things that I later regret” was revised to “When I feel rejected, I will often say things that I wish I hadn’t.” The internal consistency for the current sample was 0.84. One item from the Youth Risk Behavior Survey (“During the past 12 months, how many times were you in a physical fight?”) was used to assess frequency of involvement in a physical fight in the past 12 months (Centers for Disease Control and Prevention). YRBS is administered by the U.S. Center for Disease control to assess health-risk behaviors and includes a reliable item assessing frequency of physical fights (Li et al., 2022).

*Adolescent Peer Victimization* CA *and Peer Bullying* CA. Peer victimization and perpetration (peer bullying) was assessed using two, 2-item self-report scales with questions about frequency of peer victimization and peer bullying in and away from school on a 5-point Likert scale (King et al., 2019). The scales yielded internal consistencies of 0.56 (peer victimization) and 0.69 (peer bullying)

*Adolescent Social-Peer* CA*, Family-Parent* CA*, and School-Connectedness* CA*.* Social-peer connectedness (“How I feel about friends”) was adapted from Hemingway’s Adolescent Connectedness Scale (Karcher & Sass, 2010). Items included the following with a 5-point Likert Scale: “I have friends I’m really close to and trust completely” and “Spending time with my friends is a big part of my life.” Family-parent connectedness (“how much do people in your family understand you?”) was assessed with a 2-item measure adapted from the Parent-Family Connectedness Scale (Resnick et al., 1997) rated on a 5-point Likert scale. School connectedness (“you feel like you are a part of the school”) was assessed with a 2-item measure adapted from the 6-item School Connectedness Scale to measure youth sense of belonging in school and closeness to other classmates (Resnick et al., 1997), with response options ranging from 1 “strongly disagree” to 5 “strongly agree.” Internal consistency for these 2-item scales in the present study was 0.74, 0.76, and 0.74 for social-peer connectedness, family connectedness, and school connectedness, respectively.

*Childhood Sexual Abuse, Physical Abuse, Exposure to Violence and Life Events.* Childhood sexual and physical abuse (“People in my family have hit me so hard that it left me with bruises or marks”) were assessed with a brief two-item screener with yes/no response options. The screener originated from the Childhood trauma Questionnaire (CTQ-SF), a widely used, validated measure of childhood maltreatment that has demonstrated strong reliability (Bernstein et al., 2003; Cruz et al., 2023). Adolescents’ life events were assessed using an adapted version of The Life Events Checklist (Gray et al., 2004). Adolescents reported the presence or absence of four life events during the past 3 months, including death of a close friend/family member, relationship breakup, suspended/expelled from school or arrested, parents separated or divorced. The Life Events Checklist has demonstrated strong reliability and validity and has been used extensively in screenings for lifetime traumatic events (Gray et al., 2004; Weis et al., 2021).

**References**

Angold, A., Costello, E. J., Messer, S. C., Pickles, A., Winder, F., & Silver, D. (1995). Development of a short questionnaire for use in epidemiological studies of depression in children and adolescents. *International Journal of Methods in Psychiatric Research, 5*, 237-249.

Bernstein, D. P., Stein, J. A., Newcomb, M. D., Walker, E., Pogge, D., Ahluvalia, T., . . . Desmond, D. (2003). Development and validation of a brief screening version of the Childhood Trauma Questionnaire. *Child Abuse and Neglect, 27*(2), 169-190. doi:10.1016/S0145-2134(02)00541-0

Birmaher, B., Brent, D. A., Chiappetta, L., Bridge, J., Monga, S., & Baugher, M. (1999). Psychometric properties of the Screen for Child Anxiety Related Emotional Disorders (SCARED): a replication study. *Journal of the American Academy of Child & Adolescent Psychiatry, 38*(10), 1230-1236.

Brener, N. D., Kann, L., McManus, T., Kinchen, S. A., Sundberg, E. C., & Ross, J. G. (2002). Reliability of the 1999 youth risk behavior survey questionnaire. *Journal of adolescent health*, *31*(4), 336-342.

Burleson Daviss, W., Birmaher, B., Melhem, N. A., Axelson, D. A., Michaels, S. M., & Brent, D. A. (2006). Criterion validity of the Mood and Feelings Questionnaire for depressive episodes in clinic and non‐clinic subjects. *Journal of child psychology and psychiatry*, *47*(9), 927-934.

Cella, D., Yount, S., Rothrock, N., Gershon, R., Cook, K., Reeve, B., . . . Rose, M. (2007). The Patient-Reported Outcomes Measurement Information System (PROMIS): progress of an NIH Roadmap cooperative group during its first two years. *Medical care, 45*(5 Suppl 1), S3.

Centers for Disease Control and Prevention. (2012). National Youth Risk Behavior Survey - United States, 2011 *YRBS Questionnaire Content - 1991-2017* (Vol. 61).

Cha, M., Al-Chalabi, N., Qian, J., Chaudhary, Z., Graff, A., Gerretsen, P., ... & Deluca, V. (2023). Concordance between the Columbia-Suicide Severity Rating Scale and Beck Scale for Suicide Ideation in assessing suicide behaviour in young adults with schizophrenia spectrum disorders. *Psychiatry research*, *319*, 114965.

Chung, T., Colby, S. M., Barnett, N. P., & Monti, P. M. (2002). Alcohol Use Disorders Identification Test: Factor structure in an adolescent emergency department sample. *Alcoholism: Clinical and Experimental Research, 26*(2), 223-231.

Cruz D. (2023). Childhood Trauma Questionnaire-Short Form: Evaluation of Factor Structure and Measurement Invariance. *Journal of child & adolescent trauma*, *16*(4), 1099–1108. https://doi.org/10.1007/s40653-023-00556-8

Dugré, J. R., Giguére, C. É., Percie du Sert, O., Potvin, S., Dumais, A., & Consortium Signature (2019). The Psychometric Properties of a Short UPPS-P Impulsive Behavior Scale Among Psychiatric Patients Evaluated in an Emergency Setting. *Frontiers in psychiatry*, *10*, 139. https://doi.org/10.3389/fpsyt.2019.00139

Eisenberg, D., Hunt, J., & Speer, N. (2013). Mental health in American colleges and universities: Variation across student subgroups and across campuses. *Journal of Nervous and Mental Disease, 201*(1), 60-67. doi:10.1097/NMD.0b013e31827ab077

Gray, M. J., Litz, B. T., Hsu, J. L., & Lombardo, T. W. (2004). Psychometric properties of the life events checklist. *Assessment, 11*(4), 330-341.

Gryczynski, J., Kelly, S. M., Mitchell, S. G., Kirk, A., O'Grady, K. E., & Schwartz, R. P. (2015). Validation and performance of the Alcohol, Smoking and Substance Involvement Screening Test (ASSIST) among adolescent primary care patients. *Addiction (Abingdon, England)*, *110*(2), 240–247. https://doi.org/10.1111/add.12767

Horowitz, L. M., Bridge, J. A., Teach, S. J., Ballard, E., Klima, J., Rosenstein, D. L., . . . Pao, M. (2012). Ask Suicide-Screening Questions (ASQ): A brief instrument for the pediatric emergency department. *Archives of Pediatrics and Adolescent Medicine, 166*(12), 1170-1176. doi:10.1001/archpediatrics.2012.1276

Horowitz, L. M., Snyder, D. J., Boudreaux, E. D., He, J. P., Harrington, C. J., Cai, J., Claassen, C. A., Salhany, J. E., Dao, T., Chaves, J. F., Jobes, D. A., Merikangas, K. R., Bridge, J. A., & Pao, M. (2020). Validation of the Ask Suicide-Screening Questions for Adult Medical Inpatients: A Brief Tool for All Ages. *Psychosomatics*, *61*(6), 713–722. https://doi.org/10.1016/j.psym.2020.04.008

Kent, L., Vostanis, P., & Feehan, C. (1997). Detection of major and minor depression in children and adolescents: evaluation of the Mood and Feelings Questionnaire. *Journal of Child Psychology and Psychiatry*, *38*(5), 565-573.

King, C. A., Grupp‐Phelan, J., Brent, D., Dean, J. M., Webb, M., Bridge, J. A., ... & Pediatric Emergency Care Applied Research Network. (2019). Predicting 3‐month risk for adolescent suicide attempts among pediatric emergency department patients. *Journal of Child Psychology and Psychiatry*, *60*(10), 1055-1064.

Karcher, M. J., & Sass, D. (2010). A multicultural assessment of adolescent connectedness: Testing measurement invariance across gender and ethnicity. *Journal of Counseling Psychology, 57*(3), 274-289.

Kroenke, K., Spitzer, R. L., & Williams, J. B. W. (2001). The PHQ-9: Validity of a brief depression severity measure. *Journal of General Internal Medicine, 16*(9), 606-613.

Lloyd, E. E., Kelley, M. L., & Hope, T. (1997, April). Self-mutilation in a community sample of adolescents: Descriptive characteristics and provisional prevalence rates. Poster session presented at the annual meeting of the Society for Behavioral Medicine, New Orleans, LA.

Lloyd-Richardson, E. E., Perrine, N., Dierker, L., & Kelley, M. L. (2007). Characteristics and functions of non-suicidal self-injury in a community sample of adolescents. *Psychological Medicine, 37*(8), 1183-1192.

Li, X., Xiang, S. T., & Dong, J. (2022). The concurrence of sexual violence and physical fighting among adolescent suicide ideators and the risk of attempted suicide. *Scientific reports*, *12*(1), 5290.

Mayes, T. L., Carmody, T., Rush, A. J., Nandy, K., Emslie, G. J., Kennard, B. D., ... & Trivedi, M. H. (2023). Predicting suicidal events: A comparison of the concise health risk tracking self-report (CHRT-SR) and the Columbia suicide severity rating scale (C-SSRS). *Psychiatry research*, *326*, 115306.

Miller, J. D., Zeichner, A., & Wilson, L. F. (2012). Personality correlates of aggression: evidence from measures of the five-factor model, UPPS model of impulsivity, and BIS/BAS. *Journal of Interpersonal Violence, 27*(14), 2903-2919. doi:10.1177/0886260512438279

McNeely, J., Strauss, S. M., Wright, S., Rotrosen, J., Khan, R., Lee, J. D., & Gourevitch, M. N. (2014). Test-retest reliability of a self-administered Alcohol, Smoking and Substance Involvement Screening Test (ASSIST) in primary care patients. *Journal of substance abuse treatment*, *47*(1), 93–101. https://doi.org/10.1016/j.jsat.2014.01.007

Nock, M. K., Park, J. M., Finn, C. T., Deliberto, T. L., Dour, H. J., & Banaji, M. R. (2010). Measuring the suicidal mind: Implicit cognition predicts suicidal behavior. *Psychological Science, 21*(4), 511-517.

Posner, K., Brown, G. K., Stanley, B., Brent, D. A., Yershova, K. V., Oquendo, M. A., . . . Shen, S. (2011). The Columbia–Suicide Severity Rating Scale: Initial validity and internal consistency findings from three multisite studies with adolescents and adults. *American Journal of Psychiatry, 168*(12), 1266-1277.

Resnick, M. D., Bearman, P. S., Blum, R. W., Bauman, K. E., Harris, K. M., Jones, J., . . . Udry, J. R. (1997). Protecting adolescents from harm: Findings from the National Longitudinal Study on Adolescent Health. *Jama, 278*(10), 823-832. doi:10.1001/jama.1997.03550100049038

Ribeiro, J. D., Bender, T. W., Selby, E. A., Hames, J. L., & Joiner, T. E. (2011). Development and validation of a brief self-report measure of agitation: The Brief Agitation Measure. *Journal of personality assessment, 93*(6), 597-604.

Saunders, J. B., Aasland, O. G., Babor, T. F., De La Fuente, J. R., & Grant, M. (1993). Development of the Alcohol Use Disorders Identification Test (AUDIT): WHO collaborative project on early detection of persons with harmful alcohol consumption--II. *Addiction, 88*(6), 791-804.

Spitzer, R. L., Kroenke, K., Williams, J. B., Patient Health Questionnaire Primary Care Study Group, & Patient Health Questionnaire Primary Care Study Group. (1999). Validation and utility of a self-report version of PRIME-MD: the PHQ primary care study. *Jama*, *282*(18), 1737-1744.

Stanford, M. S., Greve, K. W., & Dickens, T. J. (1995). Irritability and impulsiveness: Relationship to self-reported impulsive aggression. *Personality and Individual Differences, 19*(5), 757-760. doi:<http://dx.doi.org/10.1016/0191-8869(95)00144-U>

Weis, C. N., Webb, E. K., Stevens, S. K., Larson, C. L., & deRoon-Cassini, T. A. (2022). Scoring the Life Events Checklist: Comparison of three scoring methods. *Psychological trauma : theory, research, practice and policy*, *14*(4), 714–720. https://doi.org/10.1037/tra0001049

Wood, A., Kroll, L., Moore, A., & Harrington, R. (1995). Properties of the mood and feelings questionnaire in adolescent psychiatric outpatients: a research note. *Journal of child psychology and psychiatry*, *36*(2), 327-334.
